# Seroprevalence of *Orientia tsutsugamushi* and *Rickettsia typhi* in water buffaloes (*Bubalus bubalis*) from Southern Thailand

**DOI:** 10.14202/vetworld.2023.1600-1604

**Published:** 2023-08-11

**Authors:** Decha Pangjai, Phirabhat Saengsawang, Kamchai Kidsin, Ngamchit Choongkittaworn, Yukio Morita, Sumalee Boonmar

**Affiliations:** 1 Department of Medical Sciences, National Institute of Health, Ministry of Public Health, Nonthaburi 11000, Thailand; 2 Akkhraratchakumari Veterinary College, Walailak University, Nakhon Si Thammarat, 80160, Thailand; 3 One Health Research Center, Walailak University, Nakhon Si Thammarat, 80160, Thailand; 4 Animal Health Section, The Eight Regional Livestock Development, Surat Thani 84000, Thailand; 5 Department of Veterinary Medicine, School of Veterinary Medicine, Azabu University, 1-17-71 Fuchinobe, Chuo-ku, Sagamihara, Kanagawa 252-5201, Japan

**Keywords:** indirect immunofluorescence assay, *Orientia tsutsugamushi*, *Rickettsia typhi*, seroprevalence, Thailand, water buffaloes

## Abstract

**Background and Aim:**

Scrub typhus and murine typhus are globally distributed zoonoses caused by the intracellular Gram-negative bacteria *Orientia tsutsugamushi* and *Rickettsia typhi*, respectively. Numerous studies have been undertaken on rickettsial illnesses in humans and animals, including arthropod vectors, in Thailand. However, the reports on the seroprevalence of antibodies to *O. tsutsugamushi* and *R. typhi* in buffaloes is extremely rare. Thus, this study aimed to estimate the seroprevalence of both rickettsial infections in water buffaloes (*Bubalus bubalis*) in Phatthalung Province, southern Thailand.

**Materials and Methods:**

From February to March 2023, a total of 156 serum samples were collected from 156 water buffaloes on 29 farms in Phatthalung province. The sera were screened for antibodies against *O. tsutsugamushi* and *R. typhi* using an indirect immunofluorescence assay.

**Results:**

The seroprevalence of antibodies against *O. tsutsugamushi* and *R. typhi* in individual water buffaloes was 4.49% (95% confidence interval [CI]: 2.19%–8.97%) and 3.85% (95% CI: 1.77%–8.14%), respectively, whereas 31% (9/29) of the herds had buffaloes with antibodies. The number of buffaloes with scrub typhus infection and ectoparasite infestation was statistically significant (p < 0.05; odds ratio = 6.25 [95% CI: 1.19–33.33]). Intriguingly, the prevalence of scrub typhus antibodies in buffaloes that were not infested with ectoparasites was much higher than those that were.

**Conclusion:**

This is the first report of *O. tsutsugamushi* and *R. typhi* antibodies in water buffalo sera in Southern Thailand. Two serum samples showed a high antibody titer against *O. tsutsugamushi*. Seroprevalence mainly occurred in non-ectoparasite-infested buffaloes, especially for *O. tsutsugamushi* antibodies. At the herd level, one-third of the studied farms showed seroprevalence. Additional research on the occurrence of these pathogens in vectors and in other animal reservoirs is necessary.

## Introduction

Scrub typhus and murine typhus are rickettsial infections caused by the bacteria *Orientia tsutsugamushi* and *Rickettsia typhi*, respectively. Both are neglected tropical zoonoses that are major causes of febrile illness worldwide [1, 2]. Furthermore, both are transmitted through bites by their arthropod vectors, mainly ticks, followed by mites and fleas [3, 4]. There are several reports on the incidence of both diseases in humans, animals, and arthropod vectors [5–9].

In Thailand, both scrub typhus and murine typhus are life threatening diseases in humans. Nevertheless, very few studies have described the occurrence of this infection in ruminants, particularly in buffaloes [10–12]. Moreover, very little is known about the seroprevalence of rickettsial infections in water buffaloes (*Bubalus bubalis*). The Food and Agriculture Organization of the United Nations has recognized the water buffalo habitat area in Phatthalung province, Thailand, as a Globally Important Agricultural Heritage System and a pastoral agro-ecosystem. The close contact between humans and water buffaloes increases the likelihood of disease transmission, especially *O. tsutsugamushi* and *R. typhi*. Both of these infections can be detected using highly sensitive and specific indirect immunofluorescence assays (IFAs) [13, 14].

Water buffalo plays an important economic and agricultural role in Thailand. Thus, the transmission of *O. tsutsugamushi* and *R. typhi* from buffaloes to humans is a concern associated with buffalo farming. Furthermore, a serological study on the prevalence of these pathogens in water buffalo has not been conducted in this area.

Thus, this study aimed to estimate the seroprevalence of antibodies against *O. tsutsugamushi* and *R. typhi* in water buffaloes in Southern Thailand using IFA.

## Materials and Methods

### Ethical approval

The animal handling, animal restraint, and blood collection procedures in this study were approved by the Walailak University Institutional Animal Care under the approval ID: WU-ACUC-66005.

### Study period and location

The study was conducted from February to March 2023. The samples were collected in Thailand’s Phatthalung province. In addition, the samples were additionally processed at the Department of Medical Sciences, National Institutes of Health (NIH) Laboratory, Ministry of Public Health, Nonthaburi, Thailand.

### Sample size and sample collection

We calculated the sample size for prevalence estimation using the Epitools program (https://epitools.ausvet.com.au/samplesize) with a 95% confidence interval (CI), 5% precision, and a previous prevalence of approximately 10% by Abanda *et al*. [15] in ruminants in Thailand. The target animals in the study area of Phatthalung province, Southern Thailand, were 3000 water buffaloes (N). The results showed that samples from at least 133 water buffaloes were required. The samples were collected using the cluster sampling technique and each water buffalo farm was considered a cluster unit. Approximately 5 mL of blood was collected from the jugular vein and placed into a plain blood collection tube. Following centrifugation at 1500 × *g* for 15 min, the separated serum samples were stored at −20°C until processing at the Department of Medical Sciences, NIH Laboratory, where the serum samples were placed into new sterile microcentrifuge tubes. The characteristics of each water buffalo, including their gender, age, health status, and the presence or absence of ectoparasite infestation, were noted.

### Immunofluorescence assay

The IFA method of Boonmar *et al*. [16] was used to test the buffalo serum for antibodies to *R. typhi* and *O. tsutsugamushi*. Briefly, *R. typhi* and *O. tsutsugamushi* antigens were placed on the test slides (courtesy of the Kanagawa Prefectural Institute of Public Health in Japan) and dried at the room temperature (30°C). Then, the antigen-fixed slide was incubated at 37°C for 1 h in a humidity chamber with 10 μL of diluted serum (diluted in phosphate-buffered saline [PBS] containing 5% skim milk) added to the test holes. Next, the slides were washed twice with PBS for 15 min. Then, 10 μL of a diluted (1:800) solution of fluorescein-conjugated goat anti-bovine immunoglobulin G (KPL Antibody and Conjugates Products, Sera Care Corp., USA) was added into each hole. Next, the fluorescently labeled slide was washed twice with PBS for 15 min and once with distilled water for 10 min. Finally, the slides were dried and examined using fluorescence microscopy. The IFA intensity indicated the presence of intracellular organisms and was scored as previously described by Saengsawang *et al*. [17], with the intensity of the bacillus-specific fluorescence subjectively graded from +1 to +4. Both positive and negative controls were used. A fluorescence score of +2 at a dilution of 1:16 was considered positive. All samples that were positive at the 1:16 dilution were titrated in a series of 2-fold dilutions up to 1:1024.

### Statistical analysis

The data were summarized using descriptive statistics. In addition, Fisher’s exact test was performed as a univariable analysis to test the difference between the positive proportions of each factor. Only significant factors from the univariable analysis were used in further calculations and the odds ratio (OR) was estimated using the “epiR” package incorporated in R v4.02 software (R Foundation for Statistical Computing, Vienna, Austria) [18]. The test that revealed p < 0.05 was considered statistically significant.

## Results

In total, 156 serum samples were collected from 156 water buffaloes on 29 farms in Phatthalung province (female: 106, 67.9%; male: 50, 32.1%). The buffaloes were grouped according to age (>2 years: 93, 59.6% and <2 years: 63, 40.4%). Ectoparasite infestations (lice) were detected in 109 (70.0%) buffaloes.

Antibodies against *O. tsutsugamushi* and *R. typhi* were detected in 4.49% (7/156; 95% CI: 2.19%–8.97%) and 3.85% (6/156; 95% CI: 1.77%–8.14%) of the serum samples, respectively, at the cutoff titer of 1:16. Table-1 presents the seroprevalence and antibody titer distribution of antibodies against *O. tsutsugamushi* and *R. typhi*. Seven water buffaloes tested positive for antibodies against *O. tsutsugamushi* (1:16, 1; 1:64, 4; and 1:128, 2) and six tested positive for antibodies against *R. typhi* (1:16, 4 and 1:32, 2).

**Table-1:** Seroprevalence of antibodies against *O. tsutsugamush*i and *R. typhi* by IFA.

Antibody against	Percentage of antibody	Antibody titers			

		16	32	64	128
*O. tsutsugamushi*	4.49 (7 of 156)	1	-	4	2
*R. typhi*	3.85 (6 of 156)	4	2	-	-

*O. tsutsugamushi*=*Orientia tsutsugamushi*, *R. typhi=Rickettsia typhi*, IFA=Immunofluorescence assay

Table-2 compares *O. tsutsugamushi* and *R. typhi* seroprevalence determined by IFA according to gender, age, and ectoparasite infestation using univariate analysis. There was a significant difference (p < 0.05) in the seroprevalence of antibodies against *O. tsutsugamushi* in non-ectoparasite-infested buffaloes compared with ectoparasite-infested buffaloes. No significant differences were found for other factors in water buffaloes seropositive for *O. tsutsugamushi*. Although the number of seropositive buffaloes with scrub typhus and ectoparasite infestation was significant [p < 0.05; OR = 6.25 (95% CI: 1.19–33.0)], no risk factors related to *R. typhi* seropositivity were identified.

**Table-2:** Statistical association between the seroprevalence of antibodies against *Orientia* *tsutsugamushi* and *Rickettsia typhi* and different factors (univariate analysis).

Risk factor	Total	*O. tsutsugamushi* (7 of 156)			*R. typhi* (6 of 156)		
	
		P	N	p-value	P	N	p-value
Gender							
Female	106	4	102	0.68	5	101	0.66
Male	50	3	47		1	49	
Age							
≤2 years	63	3	60	1	2	61	1
>2 years	93	4	89		4	89	
Ectoparasite							
Yes	109	2	107	< 0.05*	3	106	0.37
No	47	5	42		3	44	

P=Positive, N=Negative,

* p < 0.05 (odds=6.25 [95%CI = 1.19–33.33]). *O. tsutsugamushi=Orientia tsutsugamushi, R. typhi=Rickettsia typhi*

The antibody titers against *O. tsutsugamushi* and *R. typhi* for each water buffalo farm are presented in Table-3. At the herd level, 31% (9/29) of the studied farms were seropositive for at least one rickettsial infection. Six farms (20.69%) had at least one water buffalo with antibodies against *O. tsutsugamushi*, while five farms (17.24%) had at least one water buffalo with antibodies against *R. typhi*. In total, there were 13 seropositive buffaloes among the 29 farms. Farms 27 and 29 both had one buffalo with antibodies against both antigens.

**Table-3:** Herd seroprevalence and titer of antibodies against *O. tsutsugamushi* and *R. typhi* in positive buffaloes.

Farm	No. of buffaloes	No. of positive buffalo (Titer to *O. tsutsugamushi*)	No. of positive buffalo (Titer to *R. typhi*)
*1	11	-	1 (1:32)
2	6	-	-
*3	6	1 (1:16)	-
4	14	-	-
5	11	-	-
*6	12	-	1 (1:32)
7	12	-	-
8	12	-	-
9	9	-	-
10	7	-	-
*11	4	1 (1:64)	-
12	8	-	-
13	2	-	-
14	1	-	-
*15	2	-	1 (1:16)
*16	2	1 (1:64)	-
17	2	-	-
18	1	-	-
19	2	-	-
20	2	-	-
21	1	-	-
22	1	-	-
23	1	-	-
24	9	-	-
25	3	-	-
*26	3	1 (1:128)	-
**27	6	2 (1:128,1:64)	2 (1:16,1:16)
28	4	-	-
**29	2	1 (1:64)	1 (1:16)

* Positive farms containing positive water buffaloes;

** Farms with coinfection water buffaloes. *O. tsutsugamushi*=*Orientia tsutsugamushi*, *R. typhi*=*Rickettsia typhi*

## Discussion

This is the first investigation of the prevalence of antibodies to *O. tsutsugamushi* and *R. typhi* in water buffaloes. Most *O. tsutsugamushi* seroepidemiological research has involved small ruminants [6, 19] and cattle [7]. In addition, with the exception of buffaloes, most serological studies on *R. typhi* have been conducted on domestic ruminants [20]. The seroprevalence of spotted fever group (SFG) rickettsial infection in sheep, goats, and cattle in Sudan was determined by IFA as 59.3%, 60.1%, and 64.4%, respectively [11]. In our study, the seroprevalence of antibodies against *O. tsutsugamushi* and *R. typhi* in water buffaloes in Southern Thailand was found to be slightly low. However, the seropositivity of cattle in Japan was 9.6% [21], higher than that in our investigation. In contrast, a Kenyan study by Maina *et al*. [22] found a lower seroprevalence (1%) than in our study. However, it must be noted that none of these other studies evaluated water buffaloes. The distribution of arthropods is influenced by geographic, climatic, and ecological factors, and this may be responsible for the observed variations in seroprevalence [11].

Since we detected low antibody prevalence against *O. tsutsugamushi* in the group of buffaloes with ectoparasite infestation (p < 0.05), this factor appeared to be unrelated to the seroprevalence of *O. tsutsugamushi* antibodies in water buffaloes. In contrast, a Sudanese study by Eisawi *et al*. [11] discovered that cattle from semi-intensive management systems (infestation group) had a much higher prevalence of SFG antibodies than those from intensive management systems (no-infestation group). Similar to the Sudanese report by Eisawi *et al*. [11], we were also unable to detect a discernible variation in the seroprevalence of both antibodies according to the gender and age of the water buffaloes.

In Thailand, water buffaloes are used for traditional culture and agricultural activities. Therefore, there is a potential for an insect-borne disease, such as scrub or murine typhus, to spread to humans. Trombiculid mites have been identified as possible vectors for *O*. *tsutsugamushi* transmission between hosts [23], and the rat flea (*Xenopsylla cheopis*) was identified as a significant vector species of *R. typhi* [3]. In addition, some reports by Sumrandee *et al*. [10] and Galay *et al*. [12] indicate that infected arthropods may spread *Rickettsia* spp. to humans. In Thailand, ticks and lice were the most frequent ectoparasites found in water buffalo. Unfortunately, we could only collect lice from the buffaloes and we were unable to detect both pathogens in the lice. Additional epidemiological research is required to fully understand the role that ectoparasites or arthropods, like lice and ticks, play as reservoirs in that area.

In this study, only ectoparasite infestation was a risk factor for *O. tsutsugamushi*-seropositive water buffaloes, especially those that were ectoparasite-free at the time of blood collection. The ectoparasite-free water buffaloes had a higher rate of seropositivity than the ectoparasite-infested water buffaloes. Studies on the risk factors associated with seroprevalence have mostly been conducted on cattle and humans who work with cattle or buffaloes [24, 25]. It was found that people who lived in areas where buffaloes are reared could carry the mites. Larval mites are considered potential vectors for *O. tsutsugamushi* [26]. Regarding the risk factor finding in our study, *O. tsutsugamushi*-infected ectoparasites might have moved from one host to another or to the surrounding environment at the time of sampling, which would result in seropositivity in ectoparasite-free water buffaloes. Our investigation on the risk factors associated with buffaloes showing seropositivity for *O. tsutsugamushi* and *R. typhi* was lacking and requires further study. Importantly, ectoparasites in the environment should be investigated in future studies.

## Conclusion

This is the first study on *O. tsutsugamushi* and *R. typhi* antibody seroprevalence in water buffaloes from Southern Thailand. Antibodies to *O. tsutsugamushi* and *R. typhi* were detected in 4.49% and 3.85% of serum samples, respectively. The seroprevalence of antibodies against these antigens was significant among non-ectoparasite-infested buffaloes. At the herd level, the rate of seroprevalence was 31%. There were 13 seropositive buffaloes among 29 farms, with each farm having buffaloes positive for *O. tsutsugamushi* or *R. typhi* antibodies. To comprehend the hazards of this disease under the One Health concept, additional epidemiological research on the prevalence of rickettsial infection among water buffaloes, associated workers, and ectoparasites is required.

## Authors’ Contributions

DP and SB: Conceived and designed the study. SB, PS, NC, KK, and YM: Conducted the literature review and prepared tables. DP, SB, PS, NC, and KK: Drafted the manuscript. PS, SB, and YM: Critically revised the manuscript. DP: Conducted the laboratory tests. PS: Conducted statistical analysis. KK: Collected the samples. All authors have read, reviewed, and approved the final manuscript.
